# Hypnotism

**Published:** 1889-12-14

**Authors:** 


### HYPNOTISM.

"Cases treated by Hypnotism and Suggestion" is the title of a recent paper (*Lancet*) by Dr. C. L. Tuckey, which may be regarded as a supplement to his book on hypnotism, already reviewed in The Hospital. The induction of the hypnotic state is of course preliminary to the treatment by suggestion, and it appears to act by closing the channels of ordinary thought, and directing the attention of the patient exclusively to the point indicated by the operator. Dr. Tuckey observes that the "immense power of directed consciousness to influence organs and functions has been so well shown by Dr. Hack Tuke and other observers, that we may well credit it with most of the curative effects of the treatment." Among the cures reported by Dr. Tuckey is a case of insomnia; one of chronic diarrhœa, dating from the time of the Crimean War; one of paroxysmal sneezing; one of nocturnal enuresis; one of functional dysmenorrhœa; one of tabes dorsalis; one of torticollis; one of headache; one of scrivener's palsy; one of dipsomania. Now, it will be observed that most of the cases mentioned belong to the class of nervous maladies, and therefore the patients were doubtless of a nervous or highly susceptible temperament, and it is just such people who would be liable to be influenced by the suggestion, or rather, we should say, the command of anyone having the power to put them into the hypnotic slumber. Did we not, however, know that Dr. Tuckey is incapable of exaggeration, we should feel sceptical of chronic diarrhœa dating from the time of the Crimean War being cured by suggestion. No doubt there is such a complaint as mental or nervous diarrhœa, but we can scarcely imagine this prevailing every day, for the best part of a life-time, unaccompanied by some change of structure in the intestines, which certainly neither hypnotism nor suggestion would rectify. Chronic diarrhœa contracted in tropical or semi-tropical climates is usually associated with, if not depending upon, lardaceous or fatty deposit in the mucus coat and villi of the intestines. However, the patient cured of chronic diarrhœa is said to be of an "exceedingly nervous type," so it is possible, although not probable, that he may have suffered from nervous diarrhœa all his life! Whatever cures may be performed under hypnotism and suggestion the treatment can never become general. For every doctor is not fitted to hypnotise, and the majority of patients are unfitted to be hypnotised. Dr. Tuckey, a professed hypnotiser, admits that he failed in twenty-three cases out of one hundred. It was the same with mesmerism. Startling cases were reported of persons told to get well when under the mesmeric sleep, and obeying the command when awake again! But every practitioner was not able to mesmerise, and the majority of his patients could not be mesmerised. And so the practice being open, like hypnotism, to certain objections, fell into disuse. The same may be said and prophesied of hypnotism, which is really a modification of mesmerism.

